# Multi-organ denervation: a novel approach to combat cardiometabolic disease

**DOI:** 10.1038/s41440-023-01287-x

**Published:** 2023-04-24

**Authors:** Márcio Galindo Kiuchi, Revathy Carnagarin, Vance B. Matthews, Markus P. Schlaich

**Affiliations:** 1 Dobney Hypertension Centre, Medical School—Royal Perth Hospital Unit and RPH Research Foundation, The University of Western Australia, Perth, WA Australia; 2 Departments of Cardiology and Nephrology, Royal Perth Hospital, Perth, WA Australia; 3 Neurovascular Hypertension & Kidney Disease Laboratory, Baker Heart and Diabetes Institute, Melbourne, VIC Australia

**Keywords:** Renal denervation, Hepatic denervation, Hypertension, Sympathetic nerve activity

## Abstract

Cardiometabolic disorders are associated with a substantial loss in quality of life and pose a large burden on healthcare systems worldwide. Overactivation of the sympathetic nervous system has been shown to be a key player in several aspects relating to cardiometabolic disturbances. While diet- and exercise-induced approaches to help reduce weight remains the main strategy to combat metabolic disorders, this is often difficult to achieve. Current pharmacological approaches result in variable responses in different patient cohorts and long-term efficacy may be limited by medication side effects and non-adherence in the long term. There is a clear clinical need for complementary therapies to curb the burden of cardiometabolic disease. One such approach may include interventional sympathetic neuromodulation of organs relevant to cardiometabolic control. Data from sham-controlled clinical trials demonstrate the feasibility, safety and efficacy of catheter-based renal denervation. In analogy, denervation of the *common hepatic artery* is now feasible in humans and may prove to be similarly useful in modulating sympathetic overdrive directed towards the liver, pancreas and duodenum. Such a targeted multi-organ neuromodulation strategy may beneficially influence multiple aspects of the cardiometabolic disease continuum including blood pressure, glucose and lipid control.

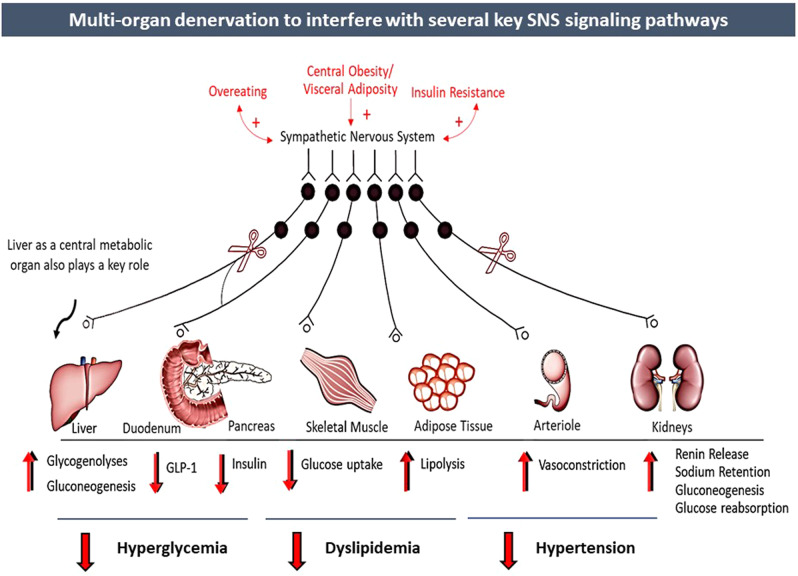

## Introduction

Cardiovascular and metabolic regulation is controlled by hormonal and neuronal signalling from the sympathetic nervous system (SNS) in relevant organs such as the kidney, liver, pancreas, skeletal muscle and adipose tissue [1–6]. SNS overactivity contributes to chronic dysregulation of cardiometabolic processes resulting in hypertension, diabetes mellitus, obesity and other disorders [7–9]. Furthermore, epidemiological and genome-wide association studies have shown disruption of the circadian rhythm in metabolic disorders, with a loss of nocturnal decline in BP which contributes to increased risk of end organ damage [10]. These metabolic disorders often co-exist and the prevalence and degree of such disorders have markedly escalated the all-cause mortality and morbidity [11–17].

The overall prevalence of hypertension in adults is around 30–45%, consistent across the world, irrespective of economic status [18, 19]. The 2016 American Diabetes Association’s (ADA’s) standards of medical care in diabetes indicate that a majority of patients with diabetes mellitus have hypertension [20, 21]. Prevalence of HTN will increase by more than 9%, or 27 million additional people, from 2010 to 2030 with annual healthcare costs of about $131 billion in the United States [22]. Current projections estimate that by 2045 over 629 million people between 20–79 years of age will have T2DM. Current global costs for treating diabetes exceed 727 billion USD per year or one out of every eight dollars spent on healthcare [23]. While there are multiple pharmacological agents available for treating the symptoms of such conditions, the lack of effective therapies that can slow down or stop the progression for these disorders makes it an urgent public health problem.

## Current standard of care and key limitations

Currently, efficient management and treatment of hypertension and diabetes demand physicians and patients’ collaboration to balance non-pharmacological and pharmacological approaches to ultimately target to prevent target organ damage and diabetic complications [16–19].

Essential or primary hypertension is currently treated with lifestyle modifications or pharmacotherapy. The initial treatment course for HTN, with Office Systolic Blood Pressure (OSBP) ≥ 130 mmHg and ≤140 mmHg, is lifestyle modifications. However, in the presence of diabetes or increased cardiovascular risk, the 2017 American College of Cardiology (ACC)/American Heart Association (AHA) guidelines recommend pharmacologic therapy for systolic BP ≥ 130 mmHg. The current guidelines recommend the use of ambulatory blood pressure monitoring (ABPM) to confirm the diagnosis of hypertension and titration of BP medications [24]. The American College of Physicians (ACP) and American Academy of Family Physicians (AAFP) have released 2018 HTN guidelines focusing on treating HTN in adults aged 60 years or older. The core recommendation for this patient population is the initiation of therapy in those with persistent systolic BP at or above 150 mmHg to achieve a target systolic BP < 150 mmHg to reduce the risk of mortality, stroke and cardiac events [24]. Of note, the ACC/AHA and ESC/ESH treatment targets are partly based on SPRINT. However, SPRINT did not include patients with diabetes.

While the clinical definition of HTN and the practice guidelines for treatment continue to evolve, the recommended treatment course is prescribing a single agent or a combination of first-line drug classes at optimal doses. This therapy includes angiotensin-converting enzyme (ACE) inhibitors or angiotensin II receptor blockers (ARBs), calcium channel blockers (CCBs) and thiazide or thiazide-like diuretics. Clinical determination of a treatment regimen is further based on the etiology of the patient’s HTN and their comorbidities such as diabetes and overall cardiovascular health. Evidence-based treatment options are more limited for the resistant HTN population where a fourth-line agent may be added, which in some cases may adversely affect metabolic control [25].

An important factor with pharmacological treatments is patient non-adherence with prescribed medication. While the exact reasons for non-adherence remain obscure, patient preference [26, 27], inconvenience of life-long therapy, pill holidays, side effects of medication and other factors are essential contributors resulting in so-called pseudo resistant HTN, which is reported in >50% of patients [28, 29]. Because of these high rates of non-adherence, it is perhaps not surprising that control rates of HTN are stagnating, and the problem is unlikely to be solved by any additional medication becoming available. In this context, where patient preference appears as an essential determinant of risk factor control, a one-time device-based approach may represent a more effective solution for these patients, provided that any relevant procedure is safe and effective.

Initial strategies in the management of T2DM includes attention to lifestyle factors (diet, weight loss, and exercise) either alone or in combination with pharmacologic treatment. Metformin is the most widely used initial pharmacologic agent to achieve glycemic goals. However, for many diabetes patients, metformin alone fails to achieve or maintain glycemic goals and is contraindicated or not tolerated. Patients then are typically treated with one or more additional oral or injected non-insulin medications such as Sodium-glucose Cotransporter-2 (SGLT-2) Inhibitors, Dipeptidyl peptidase-4 (DPP-4) inhibitors, Glucagon-like peptide-1 (GLP-1) agonists, and Thiazolidinediones (TZD) or Sulfonylureas while still on metformin.

Similar to antihypertensives, patient intolerance due to side effects of antidiabetic medications and interaction of drug regimens has been reported at 51% [17, 30, 31]. In a recent IDP meta-analysis, RAS blockers reduced the relative risk of new-onset diabetes, whereas β-blockers did not [31]. As a result of poor adherence and ineffectiveness of drugs to treat underlying causes, most T2DM patients do not reach normal plasma glucose levels and remain in a poorly controlled, pathologic haemoglobin A1C range (HbA1C levels > 9.0%) [32, 33]. The portion of the diabetic population considered well-controlled is low and has decreased in recent years from 22.5% to 17.6% [34]. Eventually, most patients require treatment intensification [35, 36] and progress into complex drug regimens with multiple daily injections [32], including insulin therapy [24, 30, 35, 37], which leads to more risk associated with hypoglycemia [38], hypoglycemia associated falls and neurological deficits [39, 40].

Although the therapeutic options discussed above are available for controlling blood glucose levels in T2DM, target blood glucose levels are not reached in most patients with current therapies. This indicates a clear need for new treatment options, specifically ones that can address the underlying causes and limit disease progression.

## The need for novel treatment approaches

While lifestyle modification and pharmacotherapy can effectively improve these disease states, non-adherence with both treatment strategies is exceedingly common and limits their effectiveness. Hence, alternative therapeutic approaches are urgently needed and ideally target pathophysiologic mechanisms relevant to these conditions. A single interventional procedure targeting sympathetic overactivity, a hallmark of these conditions, has the potential to fulfil these criteria and serve as an addition or alternative to currently available pharmacological therapies. Furthermore, the potential absence of side effects, non-adherence to medications and issues related to lifestyle modifications render this procedure an option for those patients whose target levels are not being achieved.

## Role of the sympathetic nervous system in cardiometabolic control

### Blood pressure control

**Fig. 1:**
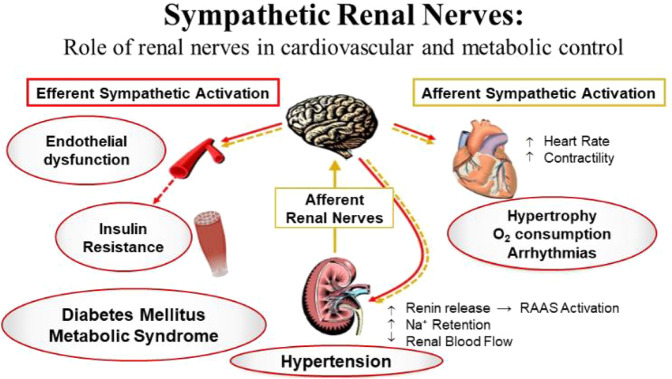
Sympathetic nervous system effects on cardiometabolic control

The integrated renal-endocrine systems that balance volume and sodium homeostasis primarily achieve blood pressure control. Renal sympathetic innervation includes efferent sympathetic fibres originating in the central nervous system and traversing to the kidney and afferent sensory fibres from the kidneys to the hypothalamus. Increased sympathetic outflow to the renal vascular bed reduces renal blood flow. It releases renin, activating the renin–angiotensin–aldosterone (RAAS) cascade, triggering vasoconstriction and enhancing tubular reabsorption of urinary sodium and water (Fig. 1). These mechanisms triggered by the efferent SNS lead to increased systemic blood volume, which circulates through constricted blood vessels, leading to system elevation of BP. In addition, the reduced renal blood flow stimulates the renal sensory nerves (afferent), resulting in an increased central SNS outflow. Both central and local increase in SNS affects many of these pathways and has been associated with HTN and its progression [41–43].

Furthermore, increased sympathetic flow leads to cardiac hypertrophy and arrhythmias, endothelial dysfunction, and insulin resistance in the skeletal muscle, contributing to the development of T2DM and the Metabolic Syndrome (MetS) [43].

### Glycaemic control

Circulating plasma glucose is derived primarily from intestinal absorption during the fed state, hepatic glycogenolysis, renal gluconeogenesis and hepatic gluconeogenesis. The liver plays a central role in maintaining blood glucose homeostasis by producing glucose following an overnight fast and releasing it to non-hepatic tissues. After food ingestion, glycogenolysis and gluconeogenesis are inhibited, and hepatic glucose uptake increases. The resulting net removal of glucose from the blood by the liver limits postprandial blood glucose elevation in the systemic circulation following a meal.

The kidneys also play a role in glucose homeostasis in the body by ensuring that glucose is not lost in the urine through three membrane protein-sodium-glucose cotransporters: SGLT1, SGLT2, and GLUT2, which are responsible for glucose reabsorption from the glomerular filtrate. Additionally, multiple studies have shown that the human kidney, through renal gluconeogenesis, releases significant amounts of glucose >50% into circulation in the postprandial state under both physiological and pathological conditions [44] and seems to be regulated by postprandial increases in sympathetic nervous system activity [45, 46].

**Fig. 2:**
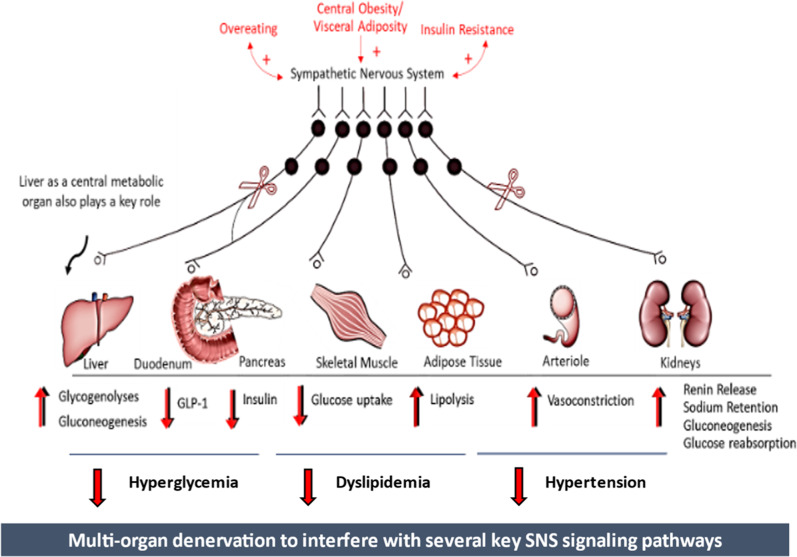
Effect of the sympathetic nervous system on cardio-metabolic homeostasis. Mechanistic overview of the potential effect of multi-organ denervation on cardiometabolism

The regulation of glucose metabolism and plasma glucose levels involves the integrated actions of multiple circulating hormones such as insulin, glucagon, catecholamines, glucose transporters and neural factors that affect various organs [47]. As a component of this regulatory complex, SNS signalling plays a fundamental role in modulating tissue metabolism and thus controlling the levels of circulating metabolites. The actions of the SNS (efferent) include stimulation of gluconeogenesis and glycogenolysis in the liver, inhibition of insulin release from pancreatic islets, inhibition of glucose uptake in skeletal muscle, lipolysis in adipose tissue, stimulation of glucose reabsorption and gluconeogenesis in kidneys, and decrease in substrate delivery to muscle as a consequence of arteriolar constriction [1] (Fig. 2).

**Fig. 3:**
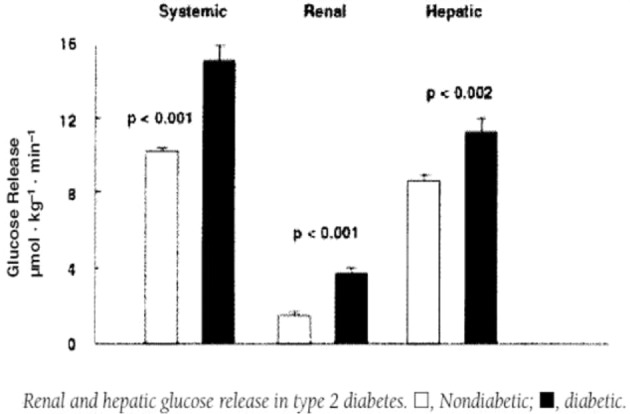
Renal and hepatic glucose release in type 2 diabetics

In the presence of T2DM and HTN, normal glucose homeostasis is disrupted. Patients with T2DM typically manifest excessive endogenous glucose production during overnight fasting, delayed suppression of endogenous glucose production following food ingestion, and impaired postprandial glucose uptake [48, 49]. Studies have demonstrated that in T2DM subjects, renal glucose release was increased by about the same extent as hepatic glucose release [41, 50] (Fig. 3). It has also been shown that renal sympathetic hyperactivity could be responsible for lower glucose uptake by peripheral tissues due to a reduction in peripheral blood flow in the presence of arterial HTN [51]. SNS-induced alterations of glucose metabolism may also be mediated via dysregulation of sodium-glucose cotransporters 2 [52]. Together, these abnormalities resulting from sympathetic overactivity in the liver and kidneys contribute to hyperglycaemia [48, 49, 53, 54].

Experimental studies have linked SNS activation or suppression in kidneys to alterations in glucose metabolism facilitated by increasing tissue glucose uptake and glycosuria induced by SGLT2 suppression and support the importance of renal SNS activation in glycaemic control [51–53]. Furthermore, addition of of empagliflozin to existing antihypertensive and antidiabetic regimen of obese older diabetic patients with uncontrolled nocturnal hypertension showed significant BP reductions [54]. SGLT2 inhibition in high sympathetic states such as obesity, high salt sensitivity, DM, nocturnal and resistant hypertension could help reduce the risk of heart failure and cardiovascular mortality, possibly due its sympatho-inhibitory effects [52–54].

### The metabolic syndrome: a marker of increased SNS activity

The MetS is a clustering of metabolic abnormalities and cardiovascular risk factors that include HTN, hyperglycaemia (prediabetes or overt T2DM), abdominal obesity, and dyslipidemia (high triglycerides and low high-density lipoproteins). The importance of liver-specific SNS signalling in regulating glucose metabolism is supported by observing a rapid and marked release of glucose from the liver following direct stimulation of hepatic sympathetic nerves [55].

**Fig. 4:**
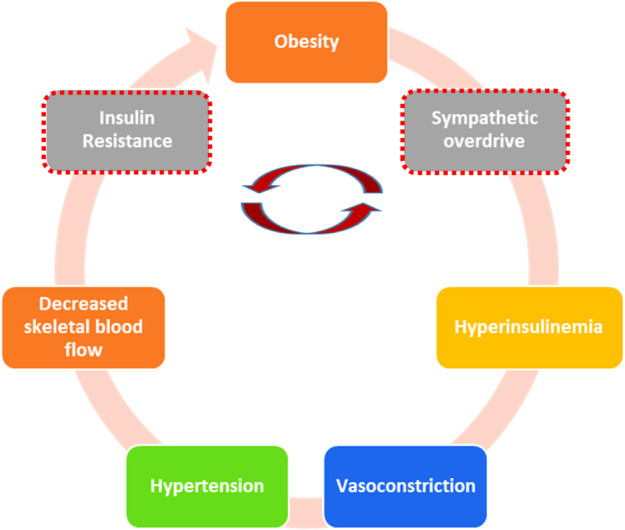
Sympathetic overdrive cycle

Multiple components of the MetS are associated with an increase in SNS activity [56]. Chronic SNS activation is higher in patients with numerous metabolic comorbidities such as HTN and T2DM [57]. Visceral fat, in particular, has been strongly linked to SNS hyperactivity [58] due to excessive adipokine release (primarily leptin). It has been suggested that once the MetS and its associated SNS hyperactivity develops, it can perpetuate into a positive feedback loop (Fig. 4), leading to a state of chronic sympathetic overdrive, causing the progression of the MetS to various chronic disorders such as HTN and T2DM [57, 59, 60].

The prevalence of the MetS was as high as 58% in a European study of patients with HTN [61] and even higher at 78% in a European study of patients with T2DM [8]. The close relationship of the MetS, HTN, T2DM, and abdominal obesity, all linked to elevated SNS overactivity, suggest that this subpopulation of patients is more likely to respond to a sympathetic denervation therapy.

## New treatment approaches

### Renal denervation

Adherence to antihypertensive medication is highly variable in clinical trials [62, 63], and non-adherence rates have been shown in up to 60% in some studies [64]. Similarly, in patients with T2DM, medication non-adherence to antidiabetic drugs may range from 53 to 65% and may be responsible for uncontrolled glycaemic levels in about 23% of cases [65]. Given these data, exploring novel therapeutic strategies that may rely less on daily adherence with prescribed medications appears appropriate. One alternative approach trialled over the last decade is catheter-based renal denervation (RDN).

This minimally invasive procedure has been reported to be safe and effective in reducing blood pressure alone in various relevant patient cohorts [64, 66–69]. Furthermore, doubts regarding its efficacy have been put aside by four recent sham-controlled randomised controlled trials [64, 66–70].

Multiple percutaneous, catheter-based renal denervation devices (Table 1) have been investigated in numerous clinical studies as a potential treatment for HTN since 2009 [71]. Recent data suggests renal denervation is a promising treatment option for HTN and is approved by The Therapeutic Goods Administration, Australia.

**Table 1:** Major renal denervation studies

Study	Type	Devices	Treated patients	Outcome
SYMPLICITY HTN-1	Prospective Cohort	Medtronic SYMPLICITY single-electrode Symplicity Flex catheter - Radiofrequency ablation	153	Persistent BP reduction after RDN, long term in patients with treatment-resistant hypertension, with good safety.
SYMPLICITY HTN-2	RCT	Medtronic SYMPLICITY single-electrode Symplicity Flex catheter - Radiofrequency ablation	52 (54 control)	Demonstrated substantial reduction in BP
SYMPLICITY HTN-3	RCT	Medtronic SYMPLICITY single-electrode Symplicity Flex catheter - Radiofrequency ablation	364 (171 control)	No overall benefit at 6 months but sustained BP reduction at 36 months
EnligHTN I	Prospective Cohort	Abbott EnligHTN multi-electrode RDN catheter ablation system that produces predictable stereotactic lesion patterns in the renal artery wall	46	Rapid and sustained BP reduction throughout 6 months
REDUCE-HTN	Prospective Cohort	BSC Vessix V2 Radiofrequency ablation	146	Premature termination – retrospect analysis showed BP reduction at 6 months and not in primary end point of 8 weeks
ACHIEVE	Prospective Cohort	Recor Paradise Endovascular ultrasound RDN system	96	Safe and sustained BP reduction through 12 months
SPYRAL HTN-OFF Med	RCT - OFF Med	Medtronic SPYRAL multielectrode SPYRAL catheter - Radiofrequency ablation	170	Demonstrated a significant difference in the primary end point in favour of RDN - 3-month change in the primary end point of 24-h ambulatory and office BPs from baseline
SPYRAL HTN-ON Med	RCT	Medtronic SPYRAL multielectrode SPYRAL catheter - Radiofrequency ablation	106	Significant drop in ABPM and safety profile
SPYRAL HTN Pivotal	Pivotal RCT	Medtronic SPYRAL multielectrode SPYRAL catheter - Radiofrequency ablation	433	Demonstarted superiority of catheter-based RDN compared with a sham procedure to safely lower blood pressure in the absence of antihypertensive medications.
Global SYMPLICITY Registry	Registry	Medtronic SPYRAL The original Symplicity Flex^TM^ renal denervation catheter or the newer-generation Symplicity Spyral^TM^ catheter	2700	BP reduction in Office BP/ ABPM were sustained to 36 months for patients who had RDN treatment with the Flex catheter
RADIANCE - SOLO	RCT - OFF Med	Recor Paradise Endovascular ultrasound RDN system	74 (72 Control)	Reduction in ABPM at 2 months in moderate HTN patients with in the absence of medications compared with sham procedure
RADIANCE TRIO	RCT	Recor Paradise Endovascular ultrasound RDN system	Approved to treat up to 146 patients	Reduction of BP at 2 months in patients with hypertension resistant to a standardised triple combination pill compared with sham procedure
RADIANCE -II	Pivotal RCT	Recor Paradise Endovascular ultrasound RDN system	Approved to treat up to 300 patients	Patients with stage II HTN, RDN resulted in a greater reduction in both systolic BP and diastolic BP at 2 months compared with a sham procedure.

Data from several registries and randomised controlled trials to evaluate RDN for HTN demonstrated the favourable safety profile of RDN and have not reported any clinically significant safety concerns. The disruption of renal nerves without affecting other abdominal, pelvic, or lower extremity innervation is supported by good long-term safety data. The Global SYMPLICITY Registry (GSR) study has enrolled over 2700 patients worldwide and reports favourable safety data for up to 3 years [72]. To date, HTN patients have been studied in various randomised sham-controlled trials of RDN, including in patients not on antihypertensive therapy without any relevant safety concerns [64, 66, 68, 70, 71, 73–75]. However, these large sham-controlled RDN trials excluded patients with an eGFR <40 ml/min/1.73 m2 and patients with type 1 diabetes or uncontrolled type 2 diabetes.

Various meta-analyses have been published to date, providing evidence of the BP-lowering effect of catheter-based denervation without significant safety concerns regarding the procedure [76]. A meta-analysis involving approximately 561 patients with resistant HTN supported the overall safety of this procedure. The adverse events identified included pseudoaneurysm at the vascular access site in 4 subjects, no evidence of significant renal artery stenosis (in 191 subjects with follow-up imaging available), and evidence for progression of previously visualised renal artery atherosclerosis in 2 subjects [77]. A more recent meta-analysis of 12 RCTs with a total of 1539 individuals reported a significant and clinically relevant reduction in 24-h BP (−4.02/−2.05 mmHg) and office BP (−8.93/−4.49 mm Hg) in uncontrolled hypertension, without increased risk of major adverse events [78].

Recently, long-term results from the SPYRAL HTN ON-MED trial showed significant reductions in 24-h systolic blood pressure and diastolic blood pressure at 24 months and 36 months in patients who underwent RDN compared with patients who underwent a sham control procedure, despite similar antihypertensive drug use, with a favourable safety profile [70]. In addition, this trial enabled the comparison of changes in blood pressure with a sham control group with similar baseline demographics and blood pressure values, showing the long-term efficacy of catheter-based renal denervation.

Initial clinical studies also yielded promising results (SYMPLICITY HTN 1-2, EnligHTN I, REDUCE-HTN, ACHIEVE). However, the SYMPLICITY HTN-3, a randomised, sham-controlled study, reported no overall treatment benefit at 6 months time point, which was attributed to two major confounding variables such as medication changes by study participants in both treatment and control arms andthe effective circumferential denervation was achieved in only a small (6%) proportion of study subjects [79]. Yet another important issue in this context is identification of the most appropriate patient cohort that may benefit from RDN. This would most likley include patient cohorts characterised by increased sympathetic activation, such as those with resistant hypertension, heart failure, chronic renal disease, and obstructive sleep apnea accompanying hypertension. However, the long term 36 month follow up of SYMPLICITY HTN-3 revealed sustained BP reductions up to 36 months clearly reflecting a benefit specific to RDN in these difficult-to-treat patients with resistant hypertension. Such a long-term FU was beneficially in overcoming [1] the Placebo – Hawthorne effect of the Sham group at 6 months and [2] the masking effect – the resistant hypertension patients in the crossover group had a consistently higher BP, despite being treated with maximally tolerated doses of antihypertensive medications at the primary endpoint at 6 months [79].

On similar grounds, the DREAMS-Study, investigating the effects of RDN on insulin sensitivity and BP in metabolic syndrome failed to show any improvement in sympathetic activity or metabolic profile [80]. This may be attributed to the small size of the cohort, lack of control group and the possibility of insufficient denervation. However, the results of the pilot study by Tsioufis et al in patients with metabolic syndrome and associated hypertension demonstrated encouraging results such as the reduction in sympathetic overdrive and restoration of normal neural response to oral glucose loading [81].

Moving forward, with knowledge of potential confounding factors and limitations encountered in the past trials, the next generation RDN devices (Medtronic SPYRAL, Recor Paradise) has been studied in the presence and absence of medication to address the confounders noted related to patient medication adherence, biases, and potential placebo effects before study expansions and randomised controlled pivotal trials. The OFF-medication trials (Table 2) have shown promise in supporting the safety and feasibility of OFF medication studies and, most importantly, the proof of principle of targeting the renal sympathetic nerves as an alternative or adjunct treatment to pharmacotherapy to reduce HTN [64, 66, 68].

**Table 2:** Blood pressure results following renal denervation ON and OFF HTN medications

Study	Mean reduction in BP compared to control	
	OSBP	24-h ASBP
SPYRAL HTN-OFF MED [66]	7.7 mm Hg	5 mm Hg
SPYRAL HTN-ON MED [64]	6.8 mm Hg	7.4 mm Hg
RADIANCE – SOLO [68]	8 mm Hg	6.3 mm Hg

Moreover, subgroup analysis of several RDN trials has also shown glycaemic and other metabolic improvements in T2DM patients [71, 73–76]. Mahfoud and colleagues investigated the effect of catheter-based RDN on glucose metabolism in a pilot study of 50 patients with HTN [73]. Three months after RDN, fasting plasma glucose (FPG) was reduced from 118 ± 3.4 to 108 ± 3.8 mg/dL (*P* = 0.039), and insulin levels were decreased from 20.8 ± 3.0 to 9.3 ± 2.5 μIU/mL (*P* = 0.006) and C peptide levels went from 5.3 ± 0.6 to 3.0 ± 0.9 ng/mL (*P* = 0.002). Experimental studies have recently shown the role of the SNS in regulating renal glucose metabolism during the development of T2DM [52, 53]. This data furthers the evidence on the role of the kidneys in T2DM and the complex pathways by which HTN and T2DM are interconnected due to the underlying central SNS overactivity.

Taken together, these data suggest that the intricate interplay between afferent and efferent signalling from organs relevant to both cardiovascular and metabolic control, such as the kidney, liver, and pancreas, may provide a unique therapeutic opportunity to selectively interrupt crucial neural signalling pathways to improve both cardiovascular and metabolic alterations. In fact, cardiometabolic neuromodulation via multi-organ sympathetic denervation could potentially provide a holistic approach to combat the cluster of metabolic abnormalities frequently encountered in patients at increased cardiometabolic risk, including those with obesity, metabolic syndrome, hypertension and T2DM.

### Hepatic denervation (HDN)

Multiple studies in experimental animals and humans have demonstrated that over-stimulation of sympathetic nerves in the liver leads to an increased hepatic release of glucose, with corresponding increased serum glucose levels [82]. Studies in dogs have shown that hepatic sympathetic denervation achieved by surgical disruption of innervation surrounding the common hepatic artery improves glucose tolerance in high fat/high fructose-fed dogs with a duration of effect of at least 3 months [83]. In addition to its glucoregulatory actions, Bruinstroop et al. demonstrated that hepatic sympathetic denervation improves the lipid profile in obese Zucker rats [84]. Of note, a recent study in mice found that diet-induced non-alcoholic fatty live disease (NAFLD), considered as the liver manifestation of the MetS, nearly doubled the firing rate of the hepatic sympathetic nerves [85] and demonstrated that denervating the hepatic sympathetic nerves reduced hepatic steatosis and improved triglyceride accumulation pathways independent of changes in body weight, caloric intake or adiposity [85]. Therefore, HDN may have an impact on sympathetically driven liver steatosis [86].

Both experimental and clinical evidence indicate that hepatic innervation is an important modulator of hepatic glucose, and that central sympathetic overactivity contributes to the pathogenesis of T2DM. Taken together, these data suggest that selective sympathetic denervation of the liver may improve metabolic function in T2DM. In addition, it is widely understood that central sympathetic overactivity plays a role in blood pressure control. Since, HDN has the potential to have an impact on reducing central sympathetic outflow through afferent pathways from the liver to the brain [87], it may influence other autonomic nervous system processes such as blood pressure control and therefore contribute to improvements in HTN.

The safety of hepatic artery denervation is supported by extensive experience with human liver transplantation, in which there is a complete disruption of innervation. Hepatic denervation following liver transplant has shown no evidence of abnormal liver function and has no significant deleterious effects on bile secretion, liver regeneration, or hepatic blood flow [88]. Regarding perfusion, the CHA only supplies approximately 10-20% of total blood flow to hepatic tissue with the portal vein suppling upwards of 80% of all hepatic blood flow [89, 90].

Procedural safety for device based HDN in humans using RF energy was assessed in the COMPLEMENT study (NCT02278068). COMPLEMENT was a first in human (FIH) feasibility study conducted in New Zealand to assess the safety and performance of hepatic sympathetic denervation for the treatment of inadequately controlled patients with T2DM.

The procedure was performed with a single point ablation catheter, where the user manually oriented the catheter and was required to perform multiple ablations. Preliminary findings through 1-year demonstrated a strong safety profile with no serious adverse device effects (SADEs) reported across 46 subjects (personal communication). Laboratory assessments of enzymes, functional and inflammatory markers showed no adverse changes in liver function, pancreatic function, kidney function through 365 days supporting safety of HDN. Vascular safety was established with follow up Computed Tomography Angiography (CTA) imaging.

Data on the clinical benefit in regards to changes in markers of glucose metabolism and other relevant outcomes are expected to be published shortly but the procedural feasibility, organ and vascular safety of percutaneous HDN seems to be established.

In summary, combined experimental evidence and preliminary COMPLEMENT data have shown procedural feasibility and safety for hepatic denervation and provide early evidence of both glycaemic and blood pressure effect on patients with HTN and T2DM post hepatic denervation.

### Multi-organ denervation (MDN)

Accumulating evidence has been described above through experimental and clinical data on the interplay between the liver, kidneys, and central mediation of overactive SNS. The close relationships among HTN, T2DM, sympathetic overactivity, and the MetS suggest that an intervention that inhibits sympathetic signalling to both these organs, constitutes a logical therapeutic approach to improve blood pressure and glycaemic control. In addition, this approach may also have a more substantial effect on centrally mediated sympathetic outflow to other organs central to metabolic and cardiovascular control, even outside the denervated organs.

Recently, we reported the feasibility, safety, and performance of a novel device-based approach for multi-organ denervation in a swine model over 30 and 90 days of follow-up. Five Yorkshire cross pigs underwent combined percutaneous denervation in the renal arteries and the common hepatic artery (CHA) with the iRF (integrated radiofrequency). Denervation System. Control animals (*n* = 3) were also studied. Specific energy doses were administered in the renal arteries and CHA. Blood was collected at 30 and 90 days. All animals had a pre-terminal procedure angiography. Tissue samples were collected for norepinephrine (NEPI) bioanalysis. Histopathological evaluation of collateral structures and tissues near the treatment sites was performed to assess treatment safety. All animals entered and exited the study in good health. No stenosis or vessel abnormalities were present. No significant changes in serum chemistry occurred. NEPI concentrations were significantly reduced in the liver (−88%, *p* = 0.005), kidneys (−78%, *p* < 0.001), pancreas (−78%, *p* = 0.018) and duodenum (−95%, *p* = 0.028) following multi-organ denervation treatment compared to control animals. Histologic findings were consistent with favourable tissue responses at 90 days follow-up. Significant and sustained denervation of the treated organs was achieved at 90 days without major safety events. Our findings demonstrate the feasibility of multi-organ denervation using a novel iRF Denervation System in a single procedure [91].

The most scientifically rigorous approach to study this clinical hypothesis is to perform denervation procedures for each organ (kidney and liver) separately and in combination without confounding medication for a limited period under rigorous medical care and oversight. The expected benefits will be improvement in both hypertension and hyperglycaemia.

## References

[1] Thorp AA, Schlaich MP (2015). Relevance of sympathetic nervous system activation in obesity and metabolic syndrome. J Diabetes Res.

[2] Schlaich M, Straznicky N, Lambert E, Lambert G (2015). Metabolic syndrome: a sympathetic disease?. Lancet Diabetes Endocrinol.

[3] Grundy SM (2008). Metabolic syndrome pandemic. Arterioscler Thromb Vasc Biol.

[4] Grundy SM, Cleeman JI, Daniels SR, Donato KA, Eckel RH, Franklin BA (2005). Diagnosis and management of the metabolic syndrome: an American Heart Association/National Heart, Lung, and Blood Institute Scientific Statement. Circulation.

[5] Lambert GW, Straznicky NE, Lambert EA, Dixon JB, Schlaich MP (2010). Sympathetic nervous activation in obesity and the metabolic syndrome-causes, consequences and therapeutic implications. Pharm Ther.

[6] Wang Y, Beydoun MA (2007). The obesity epidemic in the United States-gender, age, socioeconomic, racial/ethnic, and geographic characteristics: a systematic review and meta-regression analysis. Epidemiol Rev.

[7] Cornier MA, Dabelea D, Hernandez TL, Lindstrom RC, Steig AJ, Stob NR (2008). The metabolic syndrome. Endocr Rev.

[8] Marchesini G, Forlani G, Cerrelli F, Manini R, Natale S, Baraldi L (2004). WHO and ATPIII proposals for the definition of the metabolic syndrome in patients with Type 2 diabetes. Diabet Med.

[9] Lastra G, Syed S, Kurukulasuriya LR, Manrique C, Sowers JR (2014). Type 2 diabetes mellitus and hypertension: an update. Endocrinol Metab Clin North Am.

[10] Lemmer B, Oster H (2018). The role of circadian rhythms in the hypertension of diabetes mellitus and the metabolic syndrome. Curr Hypertens Rep.

[11] Lewington S, Clarke R, Qizilbash N, Peto R, Collins R, Prospective Studies C (2002). Age-specific relevance of usual blood pressure to vascular mortality: a meta-analysis of individual data for one million adults in 61 prospective studies. Lancet.

[12] Beckman JA, Creager MA (2016). Vascular complications of diabetes. Circ Res.

[13] Fowler MJ (2008). Microvascular and macrovascular complications of diabetes. Clin Diabetes.

[14] Fong DS, Aiello L, Gardner TW, King GL, Blankenship G, Cavallerano JD (2004). Retinopathy in diabetes. Diabetes Care.

[15] Buse JB, Wexler DJ, Tsapas A, Rossing P, Mingrone G, Mathieu C (2020). Update to: management of hyperglycemia in type 2 diabetes, 2018. A consensus report by the American Diabetes Association (ADA) and the European Association for the Study of Diabetes (EASD). Diabetes Care.

[16] Buse JB, Wexler DJ, Tsapas A, Rossing P, Mingrone G, Mathieu C (2020). Erratum. 2019 update to: management of hyperglycemia in type 2 diabetes, 2018. A consensus report by the American Diabetes Association (ADA) and the European Association for the Study of Diabetes (EASD). Diabetes Care.

[17] Davies MJ, D’Alessio DA, Fradkin J, Kernan WN, Mathieu C, Mingrone G (2018). Management of hyperglycemia in type 2 diabetes, 2018. A consensus report by the American Diabetes Association (ADA) and the European Association for the Study of Diabetes (EASD). Diabetes Care.

[18] Collaboration NCDRF. (2017). Worldwide trends in blood pressure from 1975 to 2015: a pooled analysis of 1479 population-based measurement studies with 19.1 million participants. Lancet.

[19] Chow CK, Teo KK, Rangarajan S, Islam S, Gupta R, Avezum A (2013). Prevalence, awareness, treatment, and control of hypertension in rural and urban communities in high-, middle-, and low-income countries. JAMA.

[20] American Diabetes A. (2018). Economic costs of diabetes in the U.S. in 2017. Diabetes Care.

[21] Emerging Risk Factors C, Sarwar N, Gao P, Seshasai SR, Gobin R, Kaptoge S (2010). Diabetes mellitus, fasting blood glucose concentration, and risk of vascular disease: a collaborative meta-analysis of 102 prospective studies. Lancet.

[22] Kirkland EB, Heincelman M, Bishu KG, Schumann SO, Schreiner A, Axon RN (2018). Trends in healthcare expenditures among US adults with hypertension: national estimates, 2003–2014. J Am Heart Assoc.

[23] International Diabetes Federation. IDF Diabetes atlas. 8th ed. Brussels BIDF. 2017. http://www.diabetesatlas.org/resources/2017-atlas.html.

[24] Qaseem A, Wilt TJ, Rich R, Humphrey LL, Frost J, Forciea MA (2017). Pharmacologic treatment of hypertension in adults aged 60 years or older to higher versus lower blood pressure targets: a clinical practice guideline from the American College of Physicians and the American Academy of Family Physicians. Ann Intern Med.

[25] Suter PM, Vetter W (1995). Metabolic effects of antihypertensive drugs. J Hypertens Suppl.

[26] Ross SA (2013). Breaking down patient and physician barriers to optimize glycemic control in type 2 diabetes. Am J Med.

[27] Tschanz MP, Watts SA, Colburn JA, Conlin PR, Pogach LM (2017). Overview and discussion of the 2017 VA/DoD clinical practice guideline for the management of type 2 diabetes mellitus in primary care. Fed Pract.

[28] de Oliveira-Filho AD, Costa FA, Neves SJ, de Lyra Junior DP, Morisky DE (2014). Pseudoresistant hypertension due to poor medication adherence. Int J Cardiol.

[29] Burnier M, Egan BM (2019). Adherence in hypertension. Circ Res.

[30] Chaudhury A, Duvoor C, Reddy Dendi VS, Kraleti S, Chada A, Ravilla R (2017). Clinical review of antidiabetic drugs: implications for type 2 diabetes mellitus management. Front Endocrinol (Lausanne).

[31] Nazarzadeh M, Bidel Z, Canoy D, Copland E, Wamil M, Majert J (2021). Blood pressure lowering and risk of new-onset type 2 diabetes: an individual participant data meta-analysis. Lancet.

[32] Home P, Riddle M, Cefalu WT, Bailey CJ, Bretzel RG, Del Prato S (2014). Insulin therapy in people with type 2 diabetes: opportunities and challenges?. Diabetes Care.

[33] Garcia-Perez LE, Alvarez M, Dilla T, Gil-Guillen V, Orozco-Beltran D (2013). Adherence to therapies in patients with type 2 diabetes. Diabetes Ther.

[34] Ali MK, Bullard KM, Saaddine JB, Cowie CC, Imperatore G, Gregg EW (2013). Achievement of goals in U.S. diabetes care, 1999-2010. N Engl J Med.

[35] Nichols GA, Koo YH, Shah SN (2007). Delay of insulin addition to oral combination therapy despite inadequate glycemic control: delay of insulin therapy. J Gen Intern Med.

[36] Khunti K, Wolden ML, Thorsted BL, Andersen M, Davies MJ (2013). Clinical inertia in people with type 2 diabetes: a retrospective cohort study of more than 80,000 people. Diabetes Care.

[37] Philippe J, Raccah D (2009). Treating type 2 diabetes: how safe are current therapeutic agents?. Int J Clin Pract.

[38] Swinnen SG, Hoekstra JB, DeVries JH (2009). Insulin therapy for type 2 diabetes. Diabetes Care.

[39] Intensive blood-glucose control with sulphonylureas or insulin compared with conventional treatment and risk of complications in patients with type 2 diabetes (UKPDS 33 (1998). UK Prospective Diabetes Study (UKPDS) Group. Lancet.

[40] Snell-Bergeon JK, Wadwa RP (2012). Hypoglycemia, diabetes, and cardiovascular disease. Diabetes Technol Ther.

[41] Esler M (2000). The sympathetic system and hypertension. Am J Hypertens.

[42] Grassi G, Mark A, Esler M (2015). The sympathetic nervous system alterations in human hypertension. Circ Res.

[43] Carnagarin R, Lambert GW, Kiuchi MG, Nolde JM, Matthews VB, Eikelis N (2019). Effects of sympathetic modulation in metabolic disease. Ann N. Y Acad Sci.

[44] Gerich JE, Meyer C, Woerle HJ, Stumvoll M (2001). Renal gluconeogenesis: its importance in human glucose homeostasis. Diabetes Care.

[45] Meyer C, Stumvoll M, Welle S, Woerle HJ, Haymond M, Gerich J (2003). Relative importance of liver, kidney, and substrates in epinephrine-induced increased gluconeogenesis in humans. Am J Physiol Endocrinol Metab.

[46] Gerich JE (2010). Role of the kidney in normal glucose homeostasis and in the hyperglycaemia of diabetes mellitus: therapeutic implications. Diabet Med.

[47] Alsahli M., Shrayyef MZ, Gerich, JE. Normal Glucose Homeostasis. In: Poretsky, L. (ed.) Principles of Diabetes Mellitus. Springer, Cham. 2017. 10.1007/978-3-319-18741-9_2.

[48] Weng J, Li Y, Xu W, Shi L, Zhang Q, Zhu D (2008). Effect of intensive insulin therapy on beta-cell function and glycaemic control in patients with newly diagnosed type 2 diabetes: a multicentre randomised parallel-group trial. Lancet.

[49] Rizza RA (2010). Pathogenesis of fasting and postprandial hyperglycemia in type 2 diabetes: implications for therapy. Diabetes.

[50] Carnagarin R, Kiuchi MG, Goh G, Adams L, Cohen N, Kavnoudias H (2021). Role of the sympathetic nervous system in cardiometabolic control: implications for targeted multiorgan neuromodulation approaches. J Hypertens.

[51] Rafiq K, Fujisawa Y, Sherajee SJ, Rahman A, Sufiun A, Kobori H (2015). Role of the renal sympathetic nerve in renal glucose metabolism during the development of type 2 diabetes in rats. Diabetologia.

[52] Matthews VB, Elliot RH, Rudnicka C, Hricova J, Herat L, Schlaich MP (2017). Role of the sympathetic nervous system in regulation of the sodium glucose cotransporter 2. J Hypertens.

[53] Cherrington AD (1999). Banting lecture 1997. Control of glucose uptake and release by the liver in vivo. Diabetes.

[54] Kario K, Okada K, Kato M, Nishizawa M, Yoshida T, Asano T (2018). 24-hour blood pressure-lowering effect of an SGLT-2 inhibitor in patients with diabetes and uncontrolled nocturnal hypertension: results from the randomized, placebo-controlled SACRA study. Circulation.

[55] Jarhult J, Falck B, Ingemansson S, Nobin A (1979). The functional importance of sympathetic nerves to the liver and endocrine pancreas. Ann Surg.

[56] Mancia G, Bousquet P, Elghozi JL, Esler M, Grassi G, Julius S (2007). The sympathetic nervous system and the metabolic syndrome. J Hypertens.

[57] Huggett RJ, Scott EM, Gilbey SG, Stoker JB, Mackintosh AF, Mary DA (2003). Impact of type 2 diabetes mellitus on sympathetic neural mechanisms in hypertension. Circulation.

[58] Moreira MC, Pinto IS, Mourao AA, Fajemiroye JO, Colombari E, Reis AA (2015). Does the sympathetic nervous system contribute to the pathophysiology of metabolic syndrome?. Front Physiol.

[59] Mahfoud F, Ewen S, Ukena C, Linz D, Sobotka PA, Cremers B (2013). Expanding the indication spectrum: renal denervation in diabetes. EuroIntervention.

[60] Alexander CM, Landsman PB, Grundy SM (2006). Metabolic syndrome and hyperglycemia: congruence and divergence. Am J Cardiol.

[61] Mule G, Calcaterra I, Nardi E, Cerasola G, Cottone S (2014). Metabolic syndrome in hypertensive patients: an unholy alliance. World J Cardiol.

[62] Brinker S, Pandey A, Ayers C, Price A, Raheja P, Arbique D (2014). Therapeutic drug monitoring facilitates blood pressure control in resistant hypertension. J Am Coll Cardiol.

[63] Jung O, Gechter JL, Wunder C, Paulke A, Bartel C, Geiger H (2013). Resistant hypertension? Assessment of adherence by toxicological urine analysis. J Hypertens.

[64] Kandzari DE, Bohm M, Mahfoud F, Townsend RR, Weber MA, Pocock S (2018). Effect of renal denervation on blood pressure in the presence of antihypertensive drugs: 6-month efficacy and safety results from the SPYRAL HTN-ON MED proof-of-concept randomised trial. Lancet.

[65] Giugliano D, Maiorino MI, Bellastella G, Esposito K (2019). Clinical inertia, reverse clinical inertia, and medication non-adherence in type 2 diabetes. J Endocrinol Invest.

[66] Townsend RR, Mahfoud F, Kandzari DE, Kario K, Pocock S, Weber MA (2017). Catheter-based renal denervation in patients with uncontrolled hypertension in the absence of antihypertensive medications (SPYRAL HTN-OFF MED): a randomised, sham-controlled, proof-of-concept trial. Lancet.

[67] Azizi M, Schmieder RE, Mahfoud F, Weber MA, Daemen J, Lobo MD, et al. Six-month results of treatment-blinded medication titration for hypertension control following randomization to endovascular ultrasound renal denervation or a sham procedure in the RADIANCE-HTN SOLO trial. Circulation. 2019;139:2542–2553. 10.1161/CIRCULATIONAHA.119.040451 30880441

[68] Azizi M, Schmieder RE, Mahfoud F, Weber MA, Daemen J, Davies J (2018). Endovascular ultrasound renal denervation to treat hypertension (RADIANCE-HTN SOLO): a multicentre, international, single-blind, randomised, sham-controlled trial. Lancet.

[69] Bohm M, Kario K, Kandzari DE, Mahfoud F, Weber MA, Schmieder RE (2020). Efficacy of catheter-based renal denervation in the absence of antihypertensive medications (SPYRAL HTN-OFF MED Pivotal): a multicentre, randomised, sham-controlled trial. Lancet.

[70] Mahfoud F, Kandzari DE, Kario K, Townsend RR, Weber MA, Schmieder RE (2022). Long-term efficacy and safety of renal denervation in the presence of antihypertensive drugs (SPYRAL HTN-ON MED): a randomised, sham-controlled trial. Lancet.

[71] Mahfoud F, Schlaich M, Kindermann I, Ukena C, Cremers B, Brandt MC (2011). Effect of renal sympathetic denervation on glucose metabolism in patients with resistant hypertension: a pilot study. Circulation.

[72] Mahfoud F, Bohm M, Schmieder R, Narkiewicz K, Ewen S, Ruilope L (2019). Effects of renal denervation on kidney function and long-term outcomes: 3-year follow-up from the Global SYMPLICITY Registry. Eur Heart J.

[73] Kadziela J, Warchol-Celinska E, Prejbisz A, Januszewicz A, Witkowski A, Tsioufis K (2018). Renal denervation - can we press the “ON” button again?. Postepy Kardiol Interwencyjnej.

[74] Pan T, Guo JH, Teng GJ (2015). Renal denervation: a potential novel treatment for type 2 diabetes mellitus?. Medicine (Baltim).

[75] Witkowski A, Prejbisz A, Florczak E, Kadziela J, Sliwinski P, Bielen P (2011). Effects of renal sympathetic denervation on blood pressure, sleep apnea course, and glycemic control in patients with resistant hypertension and sleep apnea. Hypertension.

[76] Ogoyama Y, Tada K, Abe M, Nanto S, Shibata H, Mukoyama M (2022). Effects of renal denervation on blood pressures in patients with hypertension: a systematic review and meta-analysis of randomized sham-controlled trials. Hypertens Res.

[77] Davis MI, Filion KB, Zhang D, Eisenberg MJ, Afilalo J, Schiffrin EL (2013). Effectiveness of renal denervation therapy for resistant hypertension: a systematic review and meta-analysis. J Am Coll Cardiol.

[78] Cheng X, Zhang D, Luo S, Qin S (2019). Effect of catheter-based renal denervation on uncontrolled hypertension: a systematic review and meta-analysis. Mayo Clin Proc.

[79] Bhatt DL, Vaduganathan M, Kandzari DE, Leon MB, Rocha-Singh K, Townsend RR (2022). Long-term outcomes after catheter-based renal artery denervation for resistant hypertension: final follow-up of the randomised SYMPLICITY HTN-3 Trial. Lancet (Lond, Engl).

[80] Verloop WL, Spiering W, Vink EE, Beeftink MMA, Blankestijn PJ, Doevendans PA (2015). Denervation of the renal arteries in metabolic syndrome. Hypertension.

[81] Tsioufis C, Dimitriadis K, Kasiakogias A, Kalos T, Liatakis I, Koutra E (2017). et al. Effects of multielectrode renal denervation on elevated sympathetic nerve activity and insulin resisitance in metabolic syndrome. J Hypertens.

[82] Hazlehurst JM, Woods C, Marjot T, Cobbold JF, Tomlinson JW (2016). Non-alcoholic fatty liver disease and diabetes. Metabolism.

[83] Kraft G, Vrba A, Scott M, Allen E, Edgerton DS, Williams PE (2019). Sympathetic denervation of the common hepatic artery lessens glucose intolerance in the fat- and fructose-fed dog. Diabetes.

[84] Bruinstroop E, Eliveld J, Foppen E, Busker S, Ackermans MT, Fliers E (2015). Hepatic denervation and dyslipidemia in obese Zucker (fa/fa) rats. Int J Obes (Lond).

[85] Hurr C, Simonyan H, Morgan DA, Rahmouni K, Young CN (2019). Liver sympathetic denervation reverses obesity-induced hepatic steatosis. J Physiol.

[86] Ferris HA, Kahn CR (2016). Unraveling the paradox of selective insulin resistance in the liver: the brain-liver connection. Diabetes.

[87] Mizuno K, Ueno Y (2017). Autonomic nervous system and the liver. Hepatol Res.

[88] Colle I, Van Vlierberghe H, Troisi R, De Hemptinne B (2004). Transplanted liver: consequences of denervation for liver functions. Anat Rec A Disco Mol Cell Evol Biol.

[89] Kan Z, Madoff DC (2008). Liver anatomy: microcirculation of the liver. Semin Interv Radio.

[90] Eipel C, Abshagen K, Vollmar B (2010). Regulation of hepatic blood flow: the hepatic arterial buffer response revisited. World J Gastroenterol.

[91] Kiuchi MG, Ganesan K, Keating J, Carnagarin R, Matthews VB, Herat LY (2021). Combined renal and common hepatic artery denervation as a novel approach to reduce cardiometabolic risk: technical approach, feasibility and safety in a pre-clinical model. Clin Res Cardiol.

